# Effect of Shading on Red Colour and Fruit Quality in Blush Pears “ANP-0118” and “ANP-0131”

**DOI:** 10.3390/plants9020206

**Published:** 2020-02-06

**Authors:** Madeleine Peavey, Ian Goodwin, Lexie McClymont, Subhash Chandra

**Affiliations:** Agriculture Victoria Research, Department of Jobs, Precincts and Regions, Tatura 3616, Victoria, Australia; ian.goodwin@agriculture.vic.gov.au (I.G.); lexie.mcclymont@agriculture.vic.gov.au (L.M.); subhash.chandra@agriculture.vic.gov.au (S.C.)

**Keywords:** anthocyanins, firmness, *Pyrus communis*, soluble solids concentration, spectrophotometer, sunlight

## Abstract

Some cultivars of *Pyrus communis* develop mature fruit with a distinctive red blush. Investigating the patterns of pear colour development in response to sunlight has implications for orchard management of these pears. The objectives of these experiments are to study the seasonal patterns of colour development and investigate the influence of shade and sunlight exposure on the red colour and harvest quality of blush pears “ANP-0118” and “ANP-0131”. Several long, medium and short shading treatments were applied at different stages of fruit development from 28 (“ANP-0131”) and 29 (“ANP-0118”) days after full bloom (DAFB) until harvests at 119 DAFB (“ANP-0118”) and 175 DAFB (“ANP-0131”). Fruits were measured every three weeks for colour parameters (a*, hue angle, chroma) and at harvest for quality parameters (fresh weight, visual assessments of percentage blush coverage and blush intensity, flesh firmness and soluble solids concentration). In the unshaded control, red colour increased during the growing season (increase in a* value and decrease in hue angle), as well as increasing in chroma value. Periods of shading during the season negatively affected red colour in both cultivars, as evidenced by significant decreases in a* value and increases in hue angle. Shaded fruits that were subsequently re-exposed to sunlight reacted with a dynamic increase in a* value and decrease in hue angle. Fruit shaded for the length of the experiment or prior to harvest had significantly lower a* values than the control at harvest. Visual assessment at harvest of percentage blush coverage and blush intensity were significantly affected by shading in both cultivars. Shading treatments applied early in the experiment had a negative effect on the fresh fruit weight of “ANP-0118”.

## 1. Introduction

Some pear cultivars (*Pyrus communis*) develop mature fruit that are fully red or have a red blush. Red colour in fruit peel is a highly marketable quality trait due to its attractiveness to consumers and link to a wide range of health benefits [1]. For this reason, achieving desirable red colour in new pear cultivars was a focus of the Australian National Pear Breeding Program (ANPBP) with the aim to reinvigorate interest from domestic and export fresh market consumers [2]. The two cultivars selected through the ANPBP that are grown commercially in Australia are “ANP-0118” and “ANP-0131” (marketed as Lanya^®^ and Ricó^®^, respectively) and are the subjects of this study.

Red colour in fruit is expressed through the presence of flavonoid pigments called anthocyanins. In pears, the two predominant anthocyanins are cyanidin-3-galactoside and cyanidin-3-arabinoside [3], which are distributed primarily within the hypodermal layer of the peel [4]. Anthocyanin synthesis in fruit is a function of internal and external factors. Improving our knowledge of these factors assists in the ability to control red colour formation in fruit and enhance market quality. Cultivar genetics is the primary foundation of a cultivar’s ability for anthocyanin synthesis [5]. This, and interactions with the physiological stage of the fruit, plays an integral role in baseline seasonal colour development patterns in fruit. In many fruit species, the accumulation of anthocyanins and the development of colour occurs with ripening and maturity [6]. However, this does not appear to be the case in some pear cultivars, with many peaking in anthocyanin content in the early to middle stages of fruit development and gradually decreasing with maturation [7,8]. Influences from external factors, such as light, temperature, tree nutrition, crop load, rootstocks and cultural management practices, can further modify the quality and quantity of accumulated anthocyanins [9].

During the development of blush pear fruits within a tree canopy, the red blush occurs on the surfaces exposed to direct sunlight [10]. Developmental patterns such as this have been linked to anthocyanin formation for the abatement of light stress in photosynthesising plant tissues [11,12]. Sunlight exposure of fruits appears to elicit two antagonistic outcomes in red colour development in pears. It is an absolute requirement for the biosynthesis of anthocyanins, or enhances it [13], yet it also plays a role in red colour loss through the degradation of anthocyanins [7,14].

Since relatively little is known about pear colour development in contrast to other fruits, investigating and understanding the pattern of colour development in response to environmental stimuli has implications for orchard management of these pears. The objectives of this experiment are to determine the seasonal patterns of colour development and investigate the influence of shade and sunlight exposure on red colour and fruit quality of the Australian blush pears “ANP-0118” and “ANP-0131”.

## 2. Results

### 2.1. Seasonal Pattern of Red Blush Development in Sunlight-Exposed Fruit

#### 2.1.1. “ANP-0118”

**Figure 1:**
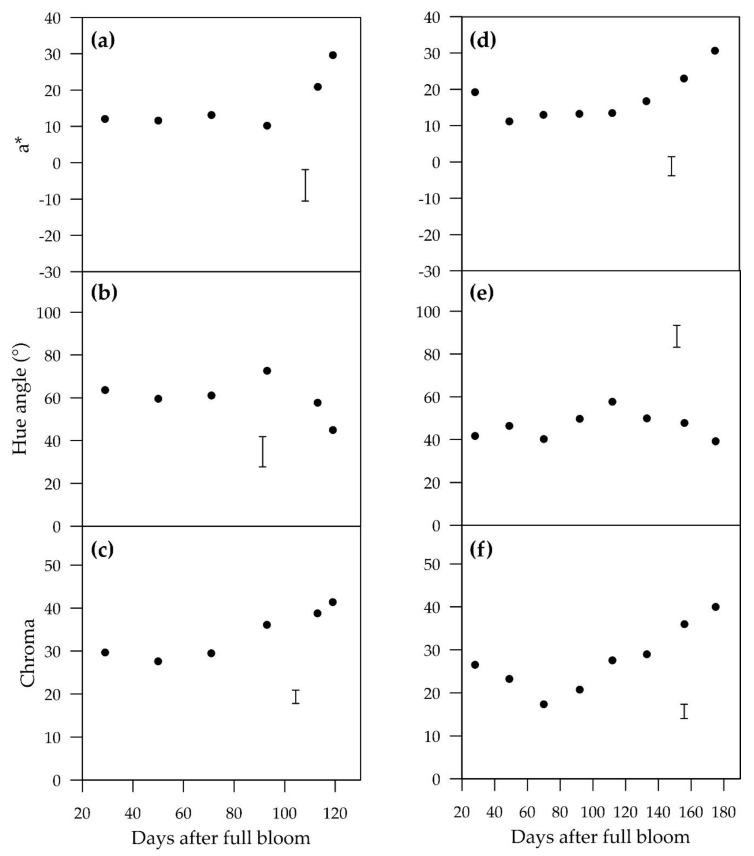
Temporal change in means of a*, hue angle and chroma of control treatments (no shading) of “ANP-0118” (**a**–**c**) and “ANP-0131” (**d**–**f**). Error bar represents Fisher’s LSD at 5% level of significance.

Red colour, represented by a* value and hue angle, made no significant developments from 29 to 93 days after full bloom (DAFB) (Figure 1a,b). Significant developments in a* and hue angle were observed from 93 DAFB until harvest at 119 DAFB. Maximum a* value (29.7) and minimum hue angle (44.9°), indicating the reddest colour, were attained at harvest. The greatest change in chroma occurred between 71 and 93 DAFB, continuing to a peak at harvest (Figure 1c).

#### 2.1.2. “ANP-0131”

At 28 DAFB, the a* value and hue angle had values of 19.2 and 41.7°, respectively, indicating that fruitlets of “ANP-0131” contained red pigment (Figure 1d,e). This was followed by a sharp drop in a* value to 11.2, a plateau from 70 to 112 DAFB, and then rapid a* development from 112 DAFB until harvest (175 DAFB). Hue angle reached a peak value of 57.7° at 112 DAFB, decreasing thereafter until harvest. The chroma value displayed a similar pattern, decreasing from 29 to 70 DAFB and thereafter increasing until harvest to a value of 36.0 (Figure 1f).

### 2.2. Effect of Shading and Fruit Growth Stage on Red Blush Colour

**Figure 2:**
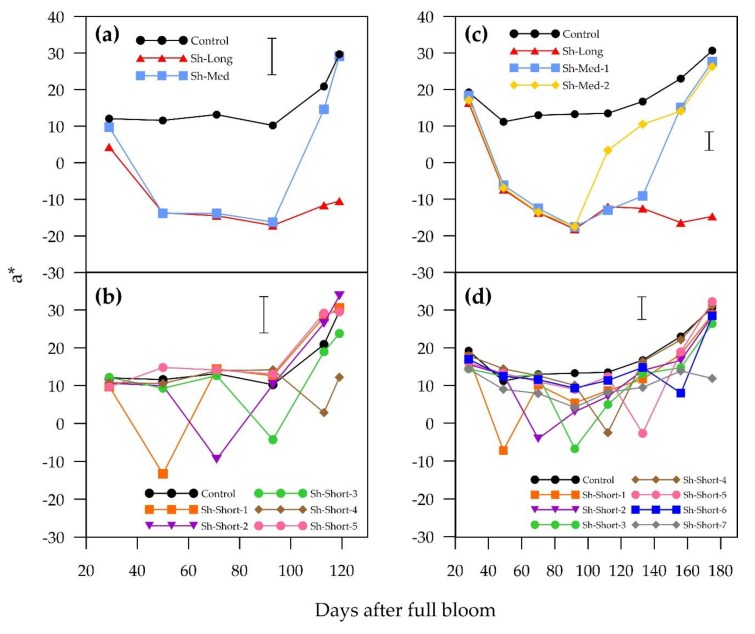
Temporal patterns of change in means of a* for “ANP-0118” (**a** = long and medium shading treatments; **b** = short shading treatments) and “ANP-0131” (**c** = long and medium shading treatments; **d** = short shading treatments). The control is displayed alongside treatments. Error bar represents Fisher’s LSD at 5% level of significance.

**Figure 3:**
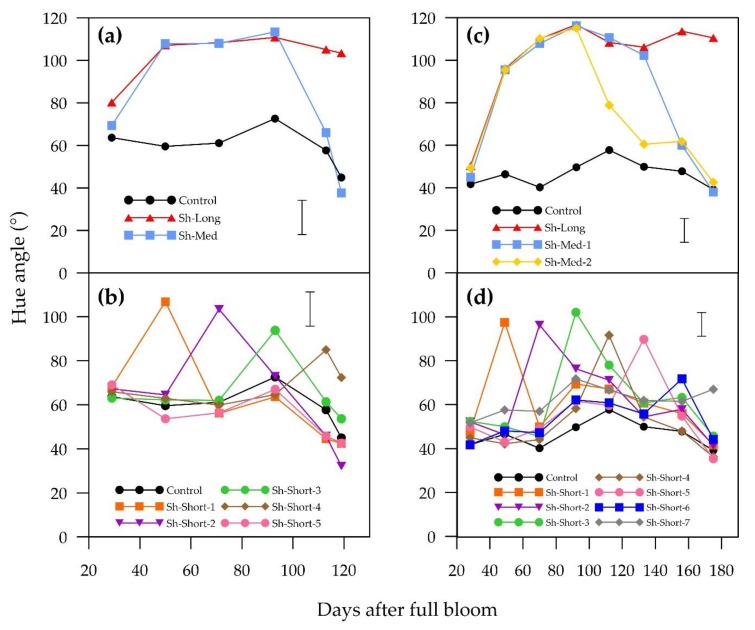
Temporal patterns of change in means of hue angle for “ANP-0118” (**a** = long and medium shading treatments; **b** = short shading treatments) and “ANP-0131” (**c** = long and medium shading treatments; **d** = short shading treatments). The control is displayed alongside treatments. Error bar represents Fisher’s LSD at 5% level of significance.

**Figure 4:**
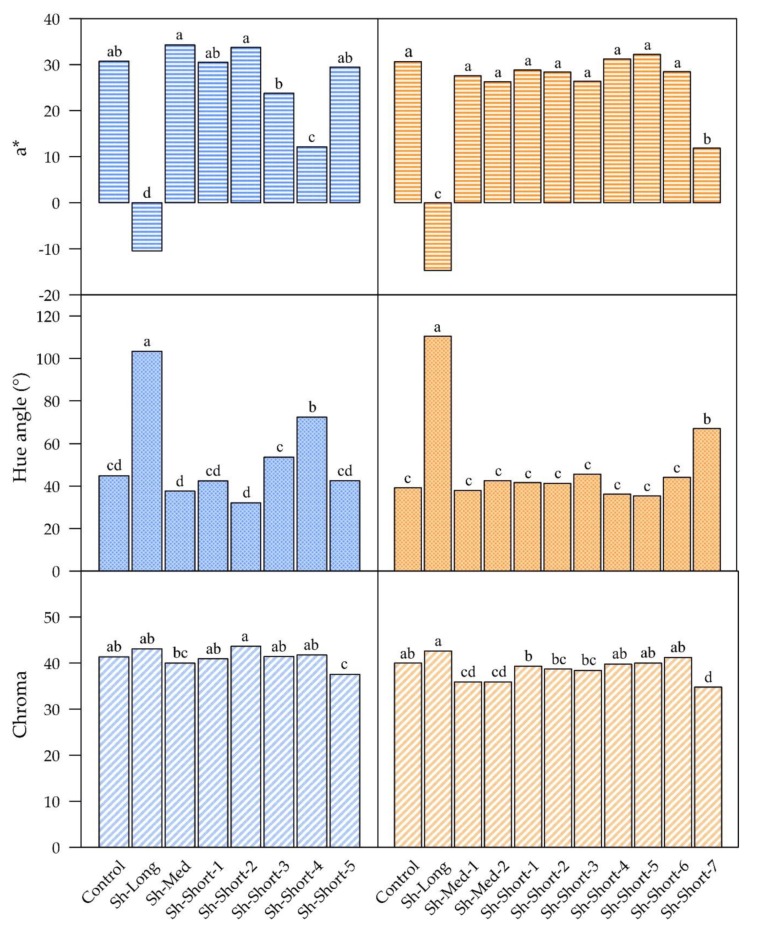
Means of a*, hue angle and chroma for “ANP-0118” at harvest (119 DAFB) and for “ANP-0131” at harvest (175 DAFB). Letters indicate means separation by Fisher’s LSD at 5% level of significance.

The interaction between the shading treatments and the growth stages was highly significant (*p* < 0.001), which implies that the temporal pattern of change in blush colour parameters was different for different shading treatments, as can be seen in Figure 2 and Figure 3. The main effects of the shading treatments and the growth stages on red colour were also highly significant (*p* < 0.001). Periods of shading in all treatments negatively affected red colour in both “ANP-0118” and “ANP-0131”, as evidenced by significant decreases in a* value and increases in hue angle, with the exception of Sh-Short-5 in “ANP-0118”. Three weeks of shading in the treatments that commenced at the beginning and middle of the experimental period resulted in an a* value below zero, indicating a complete lack of red colour and a change to green (“ANP-0118” treatments Sh-Long, Sh-Med, Sh-Short-1 to Sh-Short-3; “ANP-0131” treatments Sh-Long, Sh-Med-1, Sh-Med-2, Sh-Short-1 to Sh-Short-5). Sh-Med fruits in both cultivars, which remained shaded for longer than three weeks, maintained negative a* values and high hue angles for the remainder of their shading periods. Sh-Long in both cultivars maintained negative a* values (indicating green colour) from three weeks after commencement of shading until harvest. Earlier “ANP-0118” shading treatments that were followed by sunlight re-exposure (Sh-Med, Sh-Short-1 and Sh-Short-2) were able to recover enough red colour by harvest, as evidenced by no significant difference in a* and hue angle from the control (Figure 4). The short “ANP-0118” treatments applied afterwards (Sh-Short-3 and Sh-Short4) that allowed for more than three weeks of sunlight exposure were not able to fully recover the lost red colour by harvest. Sh-Short-5 in “ANP-0118” was not significantly different from the control. A similar pattern of colour recovery was displayed for “ANP-0131”. All treatments except for the last short treatment (Sh-Short-7) were able to sufficiently recover a* or reduce hue angle to be similar to the control at harvest (Figure 4). “ANP-0131” Sh-Short-7 treatment had significantly lower a* and higher hue angle than the control at commercial harvest, having just been shaded for three weeks.

**Figure 5:**
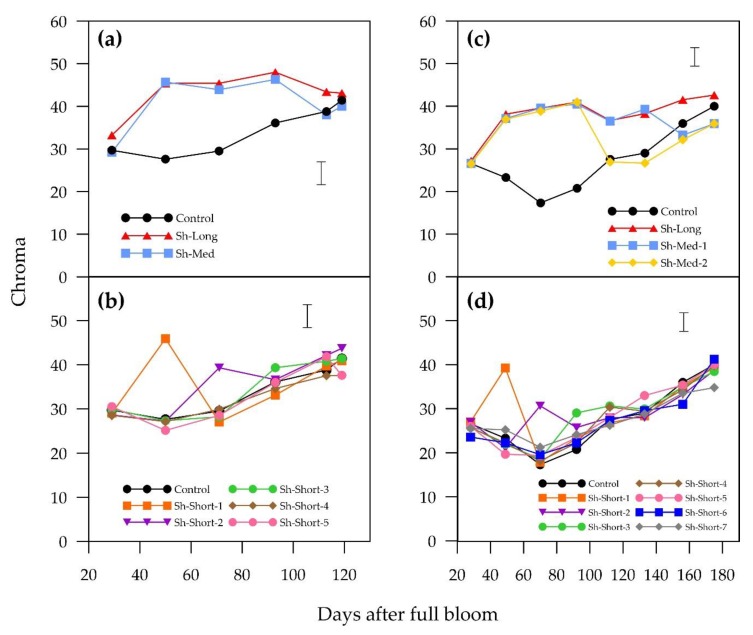
Temporal patterns of change in means of chroma for “ANP-0118” (**a** = long and medium shading treatments; **b** = short shading treatments) and “ANP-0131” (**c** = long and medium shading treatments; **d** = short shading treatments). The control is displayed alongside treatments. Error bar represents Fisher’s LSD at 5% level of significance.

The shading treatments affected blush chroma in both cultivars by increasing its value during shaded periods, except for the last “ANP-0118” short shading treatment (Sh-Short-5) which showed a decrease from 41.8 to 37.6 from 113 to 119 DAFB (Figure 5). In addition, the last two “ANP-0131” short shading treatments (Sh-Short-6 and Sh-Short-7), while not decreasing in chroma value during shading, increased their value during shading at a lower rate compared to the control and other exposed treatments. In the earlier shading treatments (“ANP-0118” treatments Sh-Med, Sh-Short-1 and Sh-Short-2; “ANP-0131” treatments Sh-Med-1, Sh-Med-2, Sh-Short-1 and Sh-Short-2), re-exposure to sunlight following shading coincided with a significant decrease in chroma value (Figure 5). Later shaded “ANP-0118” Sh-Short-3 and Sh-Short-4 fruits showed no significant changes in their chroma values in response to re-exposure at 93 and 113 DAFB, respectively. The last short shading treatments prior to harvest in both cultivars (“ANP-0118” Sh-Short-5 and “ANP-0131” Sh-Short-7) and both medium “ANP-0131” shading treatments (Sh-Med-1 and Sh_med-2) ended with a significantly lower chroma value than the control.

### 2.3. Effect of Shading on Fruit Quality Parameters at Harvest

The fruit quality parameters directly related to blush (coverage and intensity) were significantly affected by shading (Table 1 and Table 2). Fruit that had been shaded throughout the season (Sh-Long) effectively showed no blush in both cultivars. “ANP-0118” fruits that were shaded up to a month prior to harvest (Sh-Med) or late in the season (Sh-Short-4) tended to have low blush coverage and intensity (not significantly different to the control). “ANP-0131” Sh-Short-7 fruits had significantly lower blush coverage and intensity, although not to the extent of Sh-Long fruits. The lowest SSC in “ANP-0131” fruits belonged to the last short shading treatment (Sh-Short-7).

## 3. Discussion

Red colour in the cultivars in this study increased towards harvest, which is a favourable trait in terms of its marketability and attractiveness. Blush development patterns among pear cultivars are highly variable. Many cultivars, such as “Flamingo”, “Bon Rouge”, “Max Red Bartlett”, “Sensation Red Bartlett”, “Columbia Red Anjou” and “Gebhard Red Anjou”, “Red Clapp” and “Cascade”, slowly and steadily increase their hue angle over the growing season, indicating fading red colour [7,15]. Only the cultivars “Rogue Red” and “Rosired Bartlett” in the study by Dussi et al. [15] showed increasing red colour. “Forelle” and “Red Bartlett” have increasing hue angles throughout the growing season, whereas “Rosemarie” displays a highly variable hue angle in response to temperature. Since sunlight exposure and fruit peel surface temperature are linked in the field, more investigation of their combined effects on pear blush would be beneficial. The harvest hue angles for “ANP-0118” (119 DAFB) and “ANP-0131” (175 DAFB) were closer to a value of “0” (red) than those of “Rogue Red” (159 DAFB) and “Cascade” (135 DAFB), but less red than Red Clapp (124 DAFB) as stated by Dussi [10]. Hue angles of “ANP-0118” and “ANP-0131” were similar to that of “Sensation Red Bartlett” at 133 DAFB.

Sunlight exposure was a clear requirement for red colour development in the blush pears “ANP-0118” and “ANP-0131”. Fruit that were shaded for three weeks or longer either completely lost or lost a substantial amount of previously developed red colour. “ANP-0118” and “ANP-0131” fruits that were shaded for the entire experiment (Sh-Long) did not develop an a* value above zero (a* < 0 indicates green). A short shading period (six days) of “ANP-0118” fruits (Sh-Short-5) at the end of the season stalled colour development but did not result in loss of red colour.

From this it can be deduced that at this stage of fruit maturation, it takes longer than 6 days to stagnate red colour development. It was proposed by Steyn et al. [7] that continual sunlight exposure is needed to maintain anthocyanin concentrations due to dilution and degradation as the fruit grows. Their study on bagging “Rosemarie” and “Forelle” pears showed increasing anthocyanin loss with time in the absence of sunlight. In a contrasting study, “Red Bartlett” pears that were shaded and partially shaded had reduced colour fading in the month before harvest [16]. Our experiment showed that “ANP-0118” and “ANP-0131” pears that were re-exposed to sunlight, following shading and concurrent red pigment loss, entered a period of heightened pigment synthesis. This response demonstrates a strong reliance on sunlight as a stimulus for anthocyanin development in these cultivars. Anthocyanin synthesis at these times may be more centred around photoprotection since the fruits were without any protective pigment in the peel and the acuteness of the reaction upon exposure to sunlight is evident. Huang et al. [17] demonstrated this reliance on sunlight for colour development in red Chinese sand pears, with the additional factor of sunlight quantity. After a period of bagging, red Chinese sand pears that were totally re-exposed to sunlight synthesised higher amounts of anthocyanins compared to fruit re-exposed to 80% or 35% of sunlight. In our experiment, three weeks of shading prior to harvest was detrimental in both cultivars to a*, hue angle, chroma and blush intensity, emphasising the importance of fruit exposure, particularly later in the season, in achieving optimum blush. Blush coverage was severely affected in “ANP-0131” but this was not the case for “ANP-0118”, suggesting variation in colour loss mechanisms between these cultivars.

Numerous options exist to manage levels of fruit exposure. For example, vigour control by choice of rootstock and the use of deficit irrigation can be used to minimise vegetative growth, and decisions regarding tree architecture (training systems) and timing of pruning can be used to ensure good fruit exposure. However, the risks of increased fruit exposure in increasing fruit peel temperature and the incidence of sunburn need to be considered [18]. The industry would benefit from additional research into the effects of fixed and retractable shade netting on blush pear colour and other quality aspects, as they are becoming increasingly adopted to mitigate sunburn and hail damage.

The results for “ANP-0118” fresh weight suggest a relationship between timing and duration of sunlight exposure of fruit in the early stages of fruit growth and fruit size at harvest. The fruits of earlier shaded treatments (Sh-Long, Sh-Med and Sh-Short-1) finished with lower fresh weights at harvest than the control and shading treatments applied later in the season. Furthermore, the longer the fruit were shaded, the lower the fresh weight. The differences in fresh weight between treatments could indicate that early season sunlight exposure of fruit plays a role in the growth of “ANP-0118”, possibly via an impact on cell division and the final number of cells. It should be noted in this study that, although the shades were placed so that nearby leaves remained exposed, it was not possible for all leaves to be placed outside the shades. The sunlight microclimate during early growing fruit has previously been linked to fruit growth and final fruit size in “Empire” apples and “Anjou” pears [19,20]. In “Bartlett” pears, partial shading of limbs with 80% blockout from 43–138 DAFB led to a reduction in fruit growth rate, implying an effect on cell expansion. In our study, the earlier treatments that included shading from 29–50 DAFB had the most detrimental effect on final fruit diameter and weight, but perhaps sustained shading later in the season may have also reduced fruit growth rate and cell expansion.

Huang et al. [17] investigated the effects of bagging on red Chinese sand pears and observed no significant effect of bagging on SSC, which was reflected in our results for “ANP-0118”. However, while the total concentration was not affected, Huang et al. [17] reported a change in the composition of soluble sugars was observed, which was not explored in our study. “ANP-0131” fruits responded differently in that the fruit shaded in the three weeks immediately prior to harvest (Sh-Short-7) had the lowest SSC; however, there was no obvious pattern among the other values to explain the differences.

A repeat of this study may benefit from a larger sample size. Data collection performed for a subsequent growing season would very likely yield the same effect since the level of solar radiation does not tend to vary year to year.

## 4. Materials and Methods 

### 4.1. Study Site and Plant Material

Two experiments, one for “ANP-0118” (“Corella” x “Early Morettini”) and one for “ANP-0131” (“Corella” x “Doyenne du Comice”), were conducted in the Experimental Pear Orchard located at Agriculture Victoria, Tatura (Victoria, Australia; 36.439° S 145.265° E) during the 2018/2019 southern hemisphere growing season. Six-year-old trees were grafted onto BP1 rootstock and trained in a four-leader system on an Open Tatura trellis. Row and tree spacings in each plot were 4.5 and 1 m, respectively.

### 4.2. Treatments and Experimental Design

There were eight shading treatments for the “ANP-0118” experiment and eleven shading treatments for the “ANP-0131” experiment (Table 3). The shading treatments consisted of long, medium and short shading periods applied at different stages of fruit development, as well as a control treatment. Table 3 also shows the days after full bloom (DAFB) at which the experimental fruits under each shading treatment were repeatedly measured for colour parameters.

Experimental trees for “ANP-0118” and “ANP-0131” were chosen from four plots of five trees each on the western arm of the Open Tatura trellis. Fruits for experimentation were sampled from 1.2 to 1.8 m above ground level from the central three trees in each plot.

The shading treatments in each experiment were laid out in a randomised complete block design (RCBD), with the four plots in each experiment treated as blocks. For the “ANP-0118” experiment, a total of 64 fruits were chosen, with sixteen representative fruits identified in each plot and two fruits randomly assigned to each of the eight shading treatments. For the “ANP-0131” experiment, a total of 264 fruits were chosen, with 66 representative fruits identified in each plot and six fruits randomly assigned to each of the eleven shading treatments. With individual fruits used as experimental units, each shading treatment had 8 replications in the “ANP-0118” experiment, and 24 replications in the “ANP-0131” experiment. Sample fruits that were part of a cluster on a tree had the cluster thinned to only the sample fruits.

Aluminium foil umbrellas were utilised to shade the fruit from direct sunlight exposure and allow sufficient ventilation so that temperature and relative humidity were similar to ambient conditions. The control treatment consisted of fully exposed (unshaded) fruits for the duration of the experiment.

### 4.3. Measurements

Colour attributes of sample fruit were measured at approximately 3-week intervals (coinciding with the application and removal of shades) with the first measurement performed at the initiation of treatments and the last immediately postharvest. Measurements were made at the point of most intense blush located on the sunlight-exposed side of the fruit. The point of most intense blush was assessed visually at each moment of data collection to take into account blush position changes with the change of fruit position from upright (after fruit set) to hanging down as the fruit weight increased, as well as vegetative growth of the tree. Colour was assessed by the CIELAB colour scale using a portable spectrophotometer (Nix Pro, Nix Sensor Ltd., Hamilton, Ontario, Canada) with a 14 mm aperture, illuminant D65 (colour temperature 6504 K, simulates daylight), and observer angle 2°. Colour chromaticity values a*, hue angle and chroma were collected via a Bluetooth app for iOS (Nix Pro Color Sensor, Nix Sensor Ltd., version 2.6.4). Colour parameters a* and hue angle relate to the “redness” of the pear blush and chroma quantifies the colour purity or “vividness”. An a* value ≤ 0 indicates no red colour, with a maximum possible value of +60. A hue angle of 0° corresponds to red, 90° to yellow, 180° to green, and 270° to blue. Chroma values range from 0–60, with lower values being less pure (diluted with white or darkened with black) and higher values being purer. Fruit were further assessed for internal and external quality parameters immediately postharvest. Measurements of fresh weight, flesh firmness, soluble solids concentration (SSC), and visual assessments of percentage blush coverage and blush intensity were performed. All subjective visual assessments were performed by the same individual. Percentage blush coverage was evaluated for the entire fruit peel surface to the nearest 5 percent. The visual grading scale for blush intensity ranged from 1–6, with 1 being no blush and 6 being the most intense red colour. The blush intensity scales were determined for each cultivar through a sample of fruit picked at harvest time with variable blush intensities and subsequently ranked from 1 to 6. Flesh firmness and SSC measurements were all performed on the exposed cheek and the opposing unexposed cheek. Flesh firmness was measured with a penetrometer (FT327, FACCHINI srl, Alfonsine, Italy) using an 8 mm probe. SSC was measured with a digital refractometer (Model PR-1; Atago Co. Ltd., Saitama, Japan).

### 4.4. Statistical Analyses

Data on fruit characteristics measured at harvest were analysed using RCBD-based analysis of variance (ANOVA). Residual maximum likelihood (ReML) was used when there were missing data, with the effect of shading treatments treated as fixed and the effect of block and fruits within blocks as random. Data on fruit characteristics repeatedly measured over the season were analysed using RCBD-based ReML mixed models, with temporal autocorrelation accounted for using the city block metric. All analyses were conducted using Genstat^®^ 19.1 (VSN International Ltd., England, UK) statistical computing software.

## 5. Conclusions

The seasonal blush development of these cultivars differed to other pear cultivars, with red colour increasing in intensity until harvest. The results from this experiment demonstrated that the chromaticity parameters for the blush of the pear cultivars “ANP-0118” and “ANP-0131” responded in a highly dynamic manner to shading and sunlight re-exposure at all stages of fruit development. The application of shade resulted in a decrease in a* and increases in chroma and hue angle. Fruits that were re-exposed to sunlight after being shaded initially reacted with a significant increase in red colour development, at a rate faster than seasonal development, followed by a normal pattern of seasonal development. Sustained shading and later shading had the most effect on the final red colour in both cultivars. Early season shading treatments negatively affected the fresh weight of “ANP-0118” and there were significant differences between treatments in the SSC of “ANP-0131”. These results could have practical implications for the orchard management of these two cultivars, which would be honed with further research.

## Figures and Tables

**Table 1:** Means of harvest fruit quality parameters of all treatments in the “ANP-0118”. Letters indicate means separation by Fisher’s LSD (α = 0.05). Probability value (*p*) and the standard error of the means (*S.E.*) are reported.

Shade Treatment	Blush Coverage (%)		Blush Intensity (1–6)		Fresh Weight (g)		Firmness (kgf)	SSC (° Brix)
**Control**	36.2	ab	3	abc	183.6	ab	4.8	12.9
**Sh-Long**	0.3	d	1	d	131.3	c	5.3	12.8
**Sh-Med**	26.7	bc	3	c	134.1	c	5.1	12.6
**Sh-Short-1**	35.7	ab	4	ab	154.9	bc	4.9	13.0
**Sh-Short-2**	38.2	a	5	a	172.3	ab	4.8	12.8
**Sh-Short-3**	33.0	ab	4	abc	172.6	ab	5.0	13.0
**Sh-Short-4**	17.9	c	3	c	181.0	ab	4.9	13.0
**Sh-Short-5**	38.5	a	3	bc	190.1	a	4.8	12.9
***p***	<0.05		<0.05		<0.05		0.14	0.95
***S.E.***	4.01		0.50		12.7		2.01	0.39

**Table 2:** Means of harvest fruit quality parameters of all treatments in the “ANP-0131”. Letters indicate means separation by Fisher’s LSD (α = 0.05). Probability value (*p*) and the standard error of the means (S.E.) are reported.

Shade Treatment	Blush Coverage (%)		Blush Intensity (1–6)		Fresh Weight (g)	Firmness (kgf)	SSC (° Brix)	
**Control**	41.2	abcd	4	ab	231.1	5.0	13.8	ab
**Sh-Long**	0.1	f	1	e	203.5	5.1	12.9	b
**Sh-Med-1**	35.4	cd	4	ab	190.0	5.1	14.1	a
**Sh-Med-2**	36.2	bcd	4	ab	206.1	4.8	13.7	ab
**Sh-Short-1**	34.4	d	4	ab	222.9	4.9	13.5	abc
**Sh-Short-2**	38.1	abcd	4	ab	209.3	4.9	13.2	abc
**Sh-Short-3**	39.9	abcd	4	bc	209.9	5.0	13.2	abc
**Sh-Short-4**	43.8	a	5	a	208.6	4.7	14.1	a
**Sh-Short-5**	42.2	abc	5	a	213.8	5.0	14.0	a
**Sh-Short-6**	43.1	ab	4	c	193.7	4.9	13.7	ab
**Sh-Short-7**	20.4	e	3	d	202.8	5.0	12.6	c
***p***	<0.05		<0.05		0.69	0.85	<0.05	
***S.E.***	3.76		0.25		20.14	0.23	0.50	

**Table 3:** Shading treatments of blush pear cvs. “ANP-0118” and “ANP-0131” applied over the 2018/2019 growing season. ‘X’ = no shading.

	**Cv. ANP-0118**						
**Treatment**	*29–50 DAFB*	*50–71 DAFB*		*71–93 DAFB*	*93–113 DAFB*		*113–119 DAFB*
**Control**	X	X		X	X		X
**Sh-Long**	Shade	Shade		Shade	Shade		Shade
**Sh-Med**	Shade	Shade		Shade	X		X
**Sh-Short-1**	Shade	X		X	X		X
**Sh-Short-2**	X	Shade		X	X		X
**Sh-Short-3**	X	X		Shade	X		X
**Sh-Short-4**	X	X		X	Shade		X
**Sh-Short-5**	X	X		X	X		Shade
	**Cv. ANP-0131**						
	*28–49 DAFB*	*49–70 DAFB*	*70–92 DAFB*	*92–112 DAFB*	*112–133 DAFB*	*133–156 DAFB*	*156–175 DAFB*
**Control**	X	X	X	X	X	X	X
**Sh-Long**	Shade	Shade	Shade	Shade	Shade	Shade	Shade
**Sh-Med-1**	Shade	Shade	Shade	Shade	Shade	X	X
**Sh-Med-2**	Shade	Shade	Shade	X	X	X	X
**Sh-Short-1**	Shade	X	X	X	X	X	X
**Sh-Short-2**	X	Shade	X	X	X	X	X
**Sh-Short-3**	X	X	Shade	X	X	X	X
**Sh-Short-4**	X	X	X	Shade	X	X	X
**Sh-Short-5**	X	X	X	X	Shade	X	X
**Sh-Short-6**	X	X	X	X	X	Shade	X
**Sh-Short-7**	X	X	X	X	X	X	Shade
